# Amyloid-β deposition predicts oscillatory slowing of magnetoencephalography signals and a reduction of functional connectivity over time in cognitively unimpaired adults

**DOI:** 10.1093/braincomms/fcaf018

**Published:** 2025-01-20

**Authors:** Elliz P Scheijbeler, Willem de Haan, Emma M Coomans, Anouk den Braber, Jori Tomassen, Mara ten Kate, Elles Konijnenberg, Lyduine E Collij, Elsmarieke van de Giessen, Frederik Barkhof, Pieter Jelle Visser, Cornelis J Stam, Alida A Gouw

**Affiliations:** Alzheimer Center Amsterdam, Department of Neurology, Vrije Universiteit Amsterdam, Amsterdam UMC, 1081 HV Amsterdam, The Netherlands; Amsterdam Neuroscience, Neurodegeneration, 1081 HV Amsterdam, The Netherlands; Department of Clinical Neurophysiology & MEG Center, Vrije Universiteit Amsterdam, Amsterdam UMC, 1081 HV Amsterdam, The Netherlands; Alzheimer Center Amsterdam, Department of Neurology, Vrije Universiteit Amsterdam, Amsterdam UMC, 1081 HV Amsterdam, The Netherlands; Amsterdam Neuroscience, Neurodegeneration, 1081 HV Amsterdam, The Netherlands; Department of Radiology and Nuclear Medicine, Vrije Universiteit Amsterdam, Amsterdam UMC, 1081 HV Amsterdam, The Netherlands; Amsterdam Neuroscience, Brain Imaging, 1081 HV Amsterdam, The Netherlands; Alzheimer Center Amsterdam, Department of Neurology, Vrije Universiteit Amsterdam, Amsterdam UMC, 1081 HV Amsterdam, The Netherlands; Amsterdam Neuroscience, Neurodegeneration, 1081 HV Amsterdam, The Netherlands; Department of Radiology and Nuclear Medicine, Vrije Universiteit Amsterdam, Amsterdam UMC, 1081 HV Amsterdam, The Netherlands; Amsterdam Neuroscience, Brain Imaging, 1081 HV Amsterdam, The Netherlands; Department of Biological Psychology, Vrije Universiteit Amsterdam, 1081 HV Amsterdam, The Netherlands; Alzheimer Center Amsterdam, Department of Neurology, Vrije Universiteit Amsterdam, Amsterdam UMC, 1081 HV Amsterdam, The Netherlands; Amsterdam Neuroscience, Neurodegeneration, 1081 HV Amsterdam, The Netherlands; Alzheimer Center Amsterdam, Department of Neurology, Vrije Universiteit Amsterdam, Amsterdam UMC, 1081 HV Amsterdam, The Netherlands; Amsterdam Neuroscience, Neurodegeneration, 1081 HV Amsterdam, The Netherlands; Department of Radiology and Nuclear Medicine, Vrije Universiteit Amsterdam, Amsterdam UMC, 1081 HV Amsterdam, The Netherlands; Amsterdam Neuroscience, Brain Imaging, 1081 HV Amsterdam, The Netherlands; Alzheimer Center Amsterdam, Department of Neurology, Vrije Universiteit Amsterdam, Amsterdam UMC, 1081 HV Amsterdam, The Netherlands; Amsterdam Neuroscience, Neurodegeneration, 1081 HV Amsterdam, The Netherlands; Department of Radiology and Nuclear Medicine, Vrije Universiteit Amsterdam, Amsterdam UMC, 1081 HV Amsterdam, The Netherlands; Amsterdam Neuroscience, Brain Imaging, 1081 HV Amsterdam, The Netherlands; Clinical Memory Research Unit, Department of Clinical Sciences Malmö, Faculty of Medicine, Lund University, 202 13 Malmö, Sweden; Department of Radiology and Nuclear Medicine, Vrije Universiteit Amsterdam, Amsterdam UMC, 1081 HV Amsterdam, The Netherlands; Amsterdam Neuroscience, Brain Imaging, 1081 HV Amsterdam, The Netherlands; Department of Radiology and Nuclear Medicine, Vrije Universiteit Amsterdam, Amsterdam UMC, 1081 HV Amsterdam, The Netherlands; Amsterdam Neuroscience, Brain Imaging, 1081 HV Amsterdam, The Netherlands; Queen Square Institute of Neurology and Centre for Medical Image Computing, University College London, WC1N 3BG London, UK; Alzheimer Center Amsterdam, Department of Neurology, Vrije Universiteit Amsterdam, Amsterdam UMC, 1081 HV Amsterdam, The Netherlands; Amsterdam Neuroscience, Neurodegeneration, 1081 HV Amsterdam, The Netherlands; Alzheimer Center Limburg, School for Mental Health and Neuroscience, Maastricht University, 6229 ET Maastricht, The Netherlands; Department of Clinical Neurophysiology & MEG Center, Vrije Universiteit Amsterdam, Amsterdam UMC, 1081 HV Amsterdam, The Netherlands; Department of Clinical Neurophysiology & MEG Center, Vrije Universiteit Amsterdam, Amsterdam UMC, 1081 HV Amsterdam, The Netherlands

**Keywords:** Alzheimer’s disease, longitudinal, PET, neurophysiology

## Abstract

With the ongoing developments in the field of anti-amyloid therapy for Alzheimer’s disease, it is crucial to better understand the longitudinal associations between amyloid-β deposition and altered network activity in the living human brain. We included 110 cognitively unimpaired individuals (67.9 ± 5.7 years), who underwent [^18^F]flutemetamol (amyloid-β)-PET imaging and resting-state magnetoencephalography (MEG) recording at baseline and 4-year follow-up. We tested associations between baseline amyloid-β deposition and MEG measures (oscillatory power and functional connectivity). Next, we examined the relationship between baseline amyloid-β deposition and longitudinal MEG measures, as well as between baseline MEG measures and longitudinal amyloid-β deposition. Finally, we assessed associations between longitudinal changes in both amyloid-β deposition and MEG measures. Analyses were performed using linear mixed models corrected for age, sex and family. At baseline, amyloid-β deposition in orbitofrontal-posterior cingulate regions (i.e. early Alzheimer’s disease regions) was associated with higher theta (4–8 Hz) power (*β* = 0.17, *P* < 0.01) in- and lower functional connectivity [inverted Joint Permutation Entropy (JPE_inv_) theta, *β* = −0.24, *P* < 0.001] of these regions, lower whole-brain beta (13–30 Hz) power (*β* = −0.13, *P* < 0.05) and lower whole-brain functional connectivity (JPE_inv_ theta, *β* = −0.18, *P* < 0.001). Whole-brain amyloid-β deposition was associated with higher whole-brain theta power (*β* = 0.17, *P* < 0.05), lower whole-brain beta power (*β* = −0.13, *P* < 0.05) and lower whole-brain functional connectivity (JPE_inv_ theta, *β* = −0.21, *P* < 0.001). Baseline amyloid-β deposition in early Alzheimer’s disease regions also predicted future oscillatory slowing, reflected by increased theta power over time in early Alzheimer’s disease regions and across the whole brain (*β* = 0.11, *β* = 0.08, *P* < 0.001), as well as decreased whole-brain beta power over time (*β* = −0.04, *P* < 0.05). Baseline amyloid-β deposition in early Alzheimer’s disease regions also predicted a reduction in functional connectivity between these regions and the rest of the brain over time (JPE_inv_ theta, *β* = −0.07, *P* < 0.05). Baseline whole-brain amyloid-β deposition was associated with increased whole-brain theta power over time (*β* = 0.08, *P* < 0.01). Baseline MEG measures were not associated with longitudinal amyloid-β deposition. Longitudinal changes in amyloid-β deposition in early Alzheimer’s disease regions were associated with longitudinal changes in functional connectivity of early Alzheimer’s disease regions (JPE_inv_ theta, *β* = −0.19, *P* < 0.05) and the whole brain [corrected amplitude envelope correlations alpha (8–13 Hz), *β* = −0.22, *P* < 0.05]. Finally, longitudinal changes in whole-brain amyloid-β deposition were associated with longitudinal changes in whole-brain relative theta power (*β* = 0.21, *P* < 0.05). Disruptions of oscillatory power and functional connectivity appear to represent early functional consequences of emerging amyloid-β deposition in cognitively unimpaired individuals. These findings suggest a role for neurophysiology in monitoring disease progression and potential treatment effects in pre-clinical Alzheimer’s disease.

## Teaser

Longitudinal associations between early amyloid-β pathology and altered network activity in the living human brain are poorly understood. Scheijbeler *et al*. show that disruptions of magnetoencephalography signals represent early functional consequences of emerging amyloid-β deposition in cognitively unimpaired adults.

## Graphical Abstract

**Graphical Abstract:**
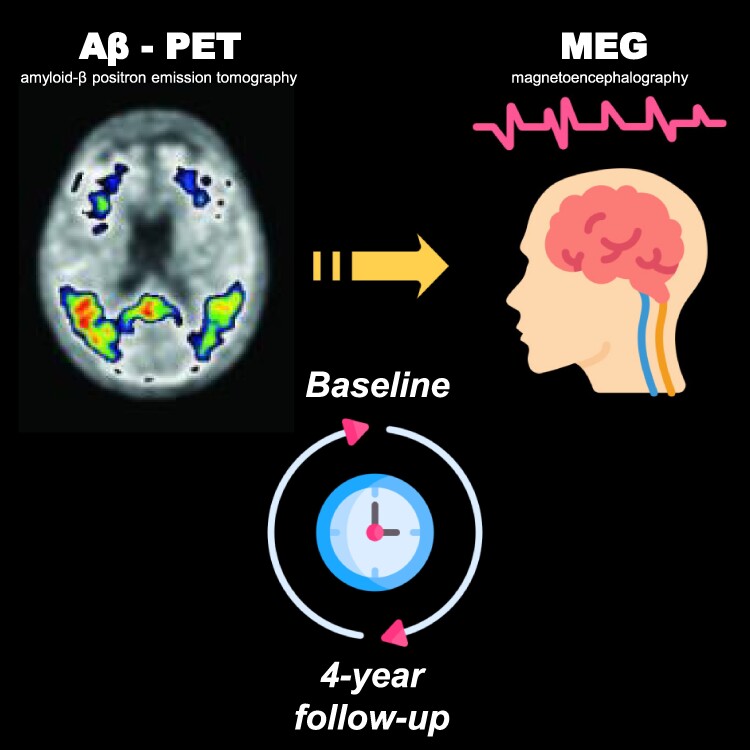

See I. Wiesman and I. Flores-Alonso (https://doi.org/10.1093/braincomms/fcaf069) for a scientific commentary on this article.

## Introduction

Alzheimer’s disease is the most common cause of dementia, with estimates of dementia due to Alzheimer’s disease at 32 million worldwide.^1^ Development of Alzheimer’s disease starts with a pre-clinical stage, in which pathological changes associated with the disease are present while cognition is still intact. Formation of neurotoxic amyloid-β (Aβ) species and accumulation of misfolded Aβ are believed to be the first pathological hallmarks of Alzheimer’s disease.^2^  *In vitro* and *in vivo* studies have demonstrated that soluble Aβ oligomers and Aβ plaques elicit abnormalities in synaptic function and neuronal network activity.^3-7^ Neuronal activity has in turn been suggested to regulate Aβ production and secretion into interstitial fluid.^4,8-12^ A major unresolved question in the field is how these cellular findings translate to those in humans. With the ongoing developments in the field of anti-amyloid therapy, it is crucial to better understand the longitudinal associations between Aβ pathology and altered network activity in the living human brain.

Studies using functional MRI (fMRI) have reported increased brain activity in individuals carrying a PSEN1 mutation up to 30 years before symptom onset.^13,14^ Neuroimaging studies combining fMRI and Aβ PET imaging have demonstrated associations between Aβ deposition and altered default mode network function in cognitively unimpaired individuals.^15-18^ fMRI relies on changes in the blood oxygen level–dependent signal as an indirect measure of neuronal activity. While Aβ-induced changes in neuronal activity could yield valuable early markers of Alzheimer’s disease, the poor temporal resolution of fMRI has limited our ability to link neurophysiological events to (progression of) Aβ pathology in humans.

Magnetoencephalography (MEG) provides a direct measurement of the synchronous activity of large groups of neurons with millisecond temporal resolution, rendering it highly advantageous for assessing neurophysiological changes in humans. Compared to EEG, MEG is less affected by conductive properties of tissues such as the skull, allowing for more precise localization of neuronal activity.^19^ Neuronal networks have the tendency to engage in oscillatory activity across multiple frequency bands.^20,21^ Oscillatory power (i.e. the level of activity within neuronal populations) and synchrony of neuronal oscillations (i.e. the level of functional connectivity between neuronal populations) are important correlates of neuronal processing that offer valuable metrics for evaluating normal and pathological brain function.^22^

Robust M/EEG findings in patients with Alzheimer’s disease include the slowing of oscillatory activity (i.e. increased power in low-frequency bands such as the delta and theta bands and decreased power in high-frequency bands such as the alpha, beta and gamma bands),^23-29^ reduced amplitude-based functional connectivity in the alpha and beta frequency bands^30-32^ and increased phase-based functional connectivity in the theta band.^31,32^ More recently, we have demonstrated that entropy-based connectivity in the theta band is reduced in prodromal Alzheimer’s disease patients compared with individuals without objective cognitive impairment.^33^ When these neurophysiological changes occur in the course of the disease, particularly in relation to the accumulation of Aβ pathology, remains unclear.

Previous multi-modal studies have combined MEG with PET imaging to better understand the relationship between Aβ pathology and neuronal dysfunction in humans. One study reported that delta-theta hyper-connectivity (as measured by imaginary coherence) co-localized with Aβ deposition in patients with prodromal or probable Alzheimer’s disease.^34^ Another study showed that greater regional Aβ burden is associated with oscillatory slowing across cortical regions in a similar study population.^35^ Two MEG-PET studies involving cognitively unimpaired individuals, or a combination of cognitively unimpaired individuals and patients with prodromal Alzheimer’s disease, demonstrated altered delta-theta phase-based connectivity in the default mode network and increased pre-frontal alpha power in Aβ-positive versus Aβ-negative individuals.^36,37^ Currently, studies including longitudinal MEG and PET measurements in the earliest stages of the disease are lacking, while these are essential to better understand the earliest amyloid-related changes in neuronal functioning.

By integrating quantitative MEG and Aβ PET measures in a unique longitudinal cohort of cognitively unimpaired individuals, this study aimed to investigate whether pre-clinical Aβ deposition is associated with altered oscillatory power and functional connectivity. In addition, we aimed to track this relationship over time. Aβ PET primarily allows for detection of aggregated forms of Aβ, such as plaques, that are known to induce abnormalities in neuronal network activity. MEG might however be able to capture subtle changes in neuronal activity associated with the presence of soluble Aβ oligomers preceding plaque formation.^38^ We hypothesized that Aβ deposition would be associated with slow oscillatory activity, reduced amplitude-based and entropy-based functional connectivity and increased phase-based functional connectivity. Aβ deposition was also expected to be associated with neurophysiological changes over time. Given that we are examining a very early stage of Aβ accumulation, we anticipated stronger effects in regions known for early Aβ deposition, i.e. the posterior cingulate cortex (PCC) and orbitofrontal gyrus (OFG),^39,40^ than across the whole brain.

## Materials and methods

### Participants

Participants were part of the ongoing longitudinal Amsterdam sub-study of the EMIF-AD PreclinAD study.^41^ This sub-study included monozygotic twin pairs, to help identify genetic and environmental pathways for Aβ pathology, other Alzheimer’s disease biomarkers and cognitive decline. Upon enrolment, all participants were ≥60 years old and exhibited normal cognition based on the performance on neuropsychological tests (for an overview of the neuropsychological testing battery, see the study by Konijnenberg et al.^41^). Exclusion criteria included any significant neurologic, systemic or psychiatric disorder that could cause cognitive impairment.^41^ For the current study, we included participants (*n* = 110, including 50 monozygotic twin pairs) for whom an Aβ PET scan, resting-state MEG recording and structural MRI scan were available at baseline and 4-year follow-up. Follow-up Aβ PET scans were collected within the AMYPAD PNHS study.^42^ This study was approved by the Medical Ethics Review Committee of the VU University Medical Center (Amsterdam, The Netherlands). All participants provided written informed consent.

### Aβ PET and MR acquisition and processing

PET scans with the Aβ binding tracer [^18^F]-flutemetamol were conducted using an Ingenuity TF PET/MRI scanner (Philips Medical Systems; time difference between longitudinal scans: 4.2 ± 0.3 years). Scans were obtained using a dynamic dual time-point acquisition method (0–30 and 90–110 min after injection of 191 ± 20 MBq [^18^F]-flutemetamol). Details on the [^18^F]-flutemetamol PET acquisition protocol are described elsewhere.^41,43-45^ Prior to each part of the dynamic scan, a T1-weighted gradient echo pulse MRI was acquired for attenuation correction purposes. First, the two parts of the dynamic scan were combined into a single multi-frame image using Vinci software (version 2.56, Max Planck Institute for Neurologic Research). Next, the isotropic structural 3D T1-weighted MR image of each individual was co-registered to the corresponding native-space PET images. Grey matter regions of interest (ROIs) of the Hammers atlas^46^ were automatically delineated on the co-registered MR images and superimposed on the [^18^F]-flutemetamol PET scans. Time activity curves were extracted using PVElab. Finally, voxel-wise parametric images of binding potentials (BP_ND_) were generated using SRTM2, as validated in previous studies.^47-49^ Cerebellar grey matter served as the reference region. We made use of continuous BP_ND_ for analysis, to provide a precise representation of the extent of Aβ deposition.

Anatomical whole brain scans were obtained using a 3.0-T Ingenuity TF PET/MR (Philips Medical Systems). Isotropic structural 3D T1-weighted images were acquired using a sagittal turbo field echo sequence (1.00 mm^3^ isotropic voxels, repetition time = 7.9 ms, echo time = 4.5 ms and flip angle = 8°).

### MEG acquisition and processing

#### Acquisition

MEG recordings were obtained in a magnetically shielded room using a 306-channel whole-head Vectorview MEG system (Elekta Neuromag Oy, Helsinki, Finland; time difference between longitudinal scans: 4.1 ± 0.3 years). The acquisition protocol consisted of two 5-min blocks of eyes-closed recording, separated by a 2-min block of recording with eyes open. Participants were in supine position and were instructed to relax but stay awake. Recordings were sampled at 1250 Hz with an online anti-aliasing filter (410 Hz) and high-pass filter (0.1 Hz). The head position relative to the MEG sensors was recorded continuously using the signals from five head position indicator coils. The head-localization coil positions and outline of the participants scalp (∼500 points) were digitized using a 3D digitizer (Fastrak, Polhemus, Colchester, VT, USA). Channels containing excessive artefacts (such as flat, very noisy and squid-jump channels) were visually identified and discarded from the raw data before applying the temporal extension of the signal space separation filter (MaxFilter software version 2.2.15 by Elekta Neuromag Oy).^50^ For the temporal extension of the signal space separation parameter settings, we set the origin to (0, 0, 40 mm) in the head coordinate frame. We used an internal expansion order of 8 and an external expansion order of 3 for the harmonic basis functions, with a correlation limit of 0.9, which was ideal for our recording site. A sliding window of 10 s was applied. The denoised signal was then reconstructed for all sensors.^51,52^ The digitized scalp surface of each participant was co-registered to the individual structural MRI using a surface matching approach. A single sphere, fitted to the outline of the scalp as obtained from the co-registered MRI, was used as a volume conductor model for the beamforming approach described below.

#### Beamforming

An atlas-based beamforming approach was used to obtain source-localized activity. For a detailed description, we refer the reader to.^53^ The broadband (0.5–100 Hz) sensor-level time series were projected through the normalized beamformer weights to reconstruct time series of neuronal activity for 90 ROIs, as included in the Automated Anatomical Labelling (AAL) atlas.^54,55^ This included 78 cortical and 12 sub-cortical regions. The source-reconstructed time series were converted to ASCII format and down-sampled to 312 Hz. For each recording, 10 non-overlapping epochs containing 4096 samples (13.1 s) of eyes-closed, artefact-free data were selected from the first eyes-closed recording, based on visual inspection by an MEG researcher (E.P.S.).

#### Oscillatory power and functional connectivity

MEG analysis was performed using BrainWave software (version 0.9.163.26, available from https://github.com/CornelisStam/BrainWave). Oscillatory power was computed for canonical frequency bands [delta (0.5–4 Hz), theta (4–8 Hz), alpha1 (8–10 Hz), alpha2 (10–13 Hz), beta (13–30 Hz) and gamma (30–48 Hz)] using a Fast Fourier Transformation. Pairwise functional connectivity was estimated using the connectivity measures listed below. All measures were estimated for each epoch separately and averaged per participant prior to group statistics.

- The joint permutation entropy (JPE) is a recently introduced functional connectivity measure that integrates information on local signal variability and interregional coupling.^33^ For a comprehensive description of the measure, as well as the role of parameter settings in entropy computations, we refer the reader to.^33^ The continuous MEG time series were first converted to a sequence of discrete symbols, with embedding dimension *n* = 4 and time-delay *τ* = 1. The joint probability of each pair of symbols was described in a matrix. Connectivity was defined as the Shannon’s information entropy of the joint probability matrix of two time series. To correct for the effects of volume conduction, the weights of pairs of identical or opposite-sign symbols in the joint probability matrix were set to zero.^56^ To simplify comparison with more conventional connectivity measures, we report inverted JPE (JPE_inv_) values, where higher values indicate stronger connectivity.
- The amplitude envelope correlation (AEC) is a measure of amplitude-based connectivity between two time series.^57-59^ The linear correlation coefficient between the amplitude envelopes of two time series was normalized to a scale from 0 to 1, where 0.5 indicates no functional connectivity. To minimize trivial spurious correlations due to volume conduction, we employed pairwise orthogonalization in both directions (*x* to *y* and *y* to *x*) before estimating the AEC.^32,57^ The AEC values (i.e. the correlation between the orthogonalized envelopes) from both directions were averaged, producing leakage-corrected AEC (AEC-c) values.
- The phase lag index (PLI) provides an estimate of phase-based connectivity between two time series.^60^ It is a measure of the asymmetry of the distribution of phase differences between two time series. Its values range between 0 and 1, with 0 indicating no connectivity and 1 referring to perfect phase locking. The measure is not affected by volume conduction, as it discards phase differences that centre around 0 mod π.

### Aβ PET and MEG ROIs

**Figure 1:**
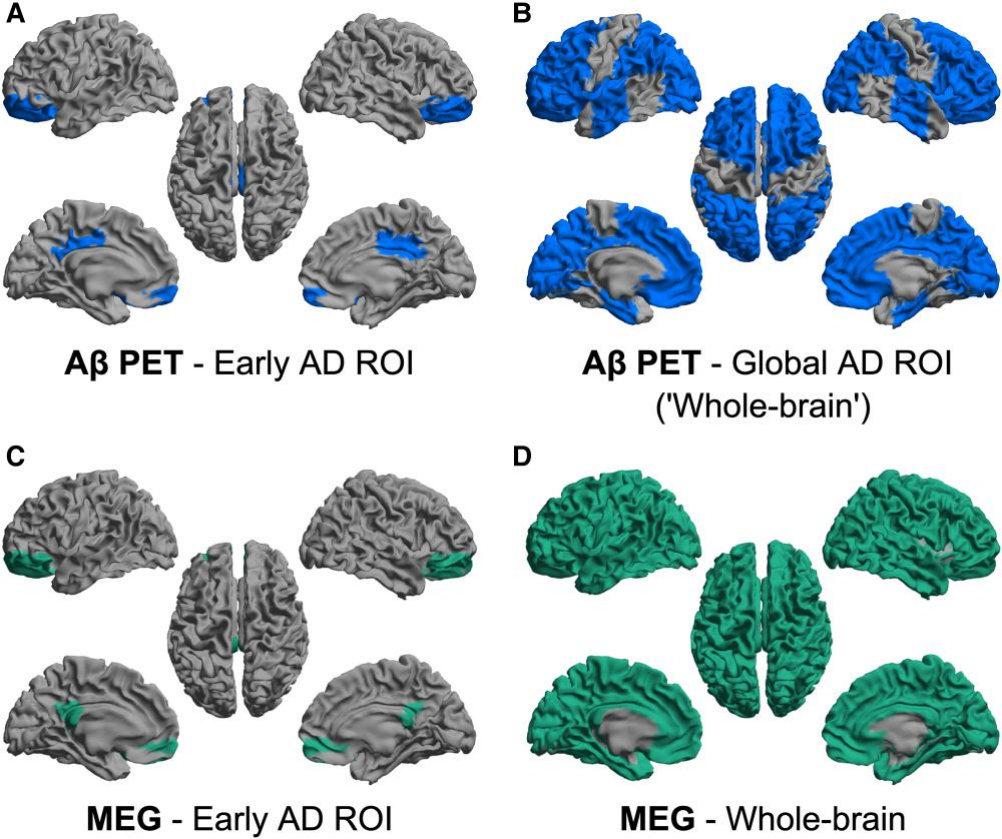
**Aβ PET and MEG ROIs.** The ROIs for Aβ PET analysis were derived from the Hammers atlas.^46^ (**A**) The early Alzheimer’s disease ROI comprised the bilateral PCC and OFG. (**B**) The global Alzheimer’s disease ROI (‘whole-brain’) included the bilateral parahippocampal gyrus, ambient gyrus, superior temporal gyrus, middle temporal gyrus, inferior temporal gyrus, fusiform gyrus, insula, anterior cingulate cortex, PCC, superior parietal gyrus, lateral parietal lobe, lateral occipital lobe, lingual gyrus, cuneus, middle frontal gyrus, gyrus rectus, OFG, inferior frontal gyrus and superior frontal gyrus. The ROIs for MEG analysis were obtained from the AAL atlas.^54,55^ (**C**) The early Alzheimer’s disease ROI, similar to PET, comprised the bilateral PCC and OFG. (**D**) Whole-brain analysis involved averaging across the 78 cortical and 12 sub-cortical regions (not visualized) delineated in the AAL atlas. Aβ, amyloid β.

Based on previous literature, we selected two composite ROIs for the Aβ PET analysis.^39,40^ First, we computed a volume-weighted average of BP_ND_ in an ‘early Alzheimer’s disease ROI’, comprising the bilateral PCC and OFG (Fig. 1A). These regions are known for exhibiting early Aβ deposition. Next, we created a ‘global Alzheimer’s disease ROI’, including all cortical regions excluding the anterior temporal lobe and the pre- and post-central gyri, known for late onset Aβ accumulation in Alzheimer’s disease^40^ (Fig. 1B). Specifically, the global Alzheimer’s disease ROI comprised the bilateral parahippocampal gyrus, ambient gyrus, superior temporal gyrus, middle temporal gyrus, inferior temporal gyrus, fusiform gyrus, insula, anterior cingulate cortex, PCC, superior parietal gyrus, lateral parietal lobe, lateral occipital lobe, lingual gyrus, cuneus, middle frontal gyrus, gyrus rectus, OFG, inferior frontal gyrus and superior frontal gyrus. By excluding regions that are known to show little to no Aβ deposition in cognitively unimpaired individuals, we improve the signal-to-noise ratio, thereby enhancing sensitivity to detect subtle changes associated with pre-clinical Alzheimer’s disease. To improve readability, we will henceforth use the term ‘whole-brain’ when referring to the global Alzheimer’s disease ROI.

For MEG analysis, we computed oscillatory power within the early Alzheimer’s disease ROI and the average functional connectivity between the early Alzheimer’s disease ROI and the rest of the brain (Fig. 1C). In addition, oscillatory power and functional connectivity were computed at whole-brain level, averaging across 78 cortical and 12 sub-cortical regions delineated in the AAL atlas^54,55^ (Fig. 1D). We chose not to exclude specific regions from MEG analysis, as alterations in brain function may extend beyond regions traditionally associated with Aβ pathology.

### Primary and secondary MEG measures

Primary MEG measures of this study included relative theta power and functional connectivity measured by JPE_inv_ theta. Relative theta power is recognized as the most potent neurophysiological biomarker of pre-dementia Alzheimer’s disease to date.^61,62^ The potential of JPE_inv_ theta connectivity as a biomarker for early-stage Alzheimer’s disease has been demonstrated more recently, with classification performance (prodromal Alzheimer’s disease patients versus controls) at least as good as the relative theta power benchmark.^33^ It is unknown whether findings from M/EEG studies in patients with subjective cognitive decline, mild cognitive impairment or Alzheimer’s disease dementia can be translated to cognitively unimpaired individuals and consequently whether we can anticipate similar characteristics in these subjects. To ensure a comprehensive assessment of the data, we therefore also evaluated relative power in five other frequency bands (i.e. delta, alpha1, alpha2, beta and gamma) as well as AEC-c alpha and PLI theta connectivites (building upon prior studies on the reproducibility of connectivity metrics^31,32^).

### Statistical analysis

We used R version 4.2.1 for statistical analyses. Linear mixed effects models (LMMs) were performed separately and independently for each MEG outcome measure (*n* = 9) and level of regional analysis [*n* = 3; (i) Aβ BP_ND_ and MEG measures in the early Alzheimer’s disease ROI, ii) Aβ BP_ND_ in the early Alzheimer’s disease ROI and whole-brain MEG measures and iii) whole-brain Aβ BP_ND_ and MEG measures]. Details of the LMM analyses can be found in the Supplementary material. First, we estimated the effect of baseline Aβ BP_ND_ on MEG measures at baseline and over time. Time was included as a continuous variable in years. An interaction term Aβ BP_ND_ × time was included to evaluate whether the relationship between baseline Aβ BP_ND_ and MEG measures changed over time. Next, we estimated the effect of baseline MEG measures on Aβ BP_ND_ at baseline and over time. Similar to before, time was included as a continuous variable in years, and an interaction term MEG measure × time was included to evaluate whether the relationship between baseline MEG measures and Aβ BP_ND_ changed over time. If the interaction term was not significant, cross-sectional associations were estimated using a LMM without interaction term. All models included subject-specific random intercepts and a random effect for family, thereby correcting for clustering in the data, and were adjusted for age and sex. The residuals in some of the LMMs did not follow a normal distribution. To ensure the robustness of our findings against potential violations of normality, we supplemented the LMMs with a non-parametric permutation approach. Specifically, we performed 1000 permutations, randomly shuffling the Aβ BP_ND_ values across individuals, while preserving the MEG measures and covariates. We conducted the permutation procedure twice, creating two separate sets of permuted datasets: one in which the whole-brain Aβ BP_ND_ values were shuffled and another in which the early Alzheimer’s disease ROI Aβ BP_ND_ values were shuffled. Using the permuted datasets, we built null distributions of the *t*-statistic for the relevant main effect (Aβ BP_ND_) or interaction effect (Aβ BP_ND_ × time) in each analysis. We then calculated the probability (*P*-value) of obtaining the *t*-statistic observed in the original models. Finally, we investigated the association between the annual change in Aβ BP_ND_ and annual change in MEG measures in our cohort using LMMs. Annual change was calculated by subtracting the baseline Aβ BP_ND_ or MEG measure from the follow-up Aβ BP_ND_ or MEG measure and dividing by the individual’s follow-up time. Again, the LMMs accounted for familial clustering and were adjusted for age and sex. We scaled predictor and outcome variables within each LMM to enable comparison of effect sizes. A *P*-value <0.05 was considered significant. For primary MEG outcome measures (relative theta power and JPE_inv_ theta connectivity), both uncorrected *P*-values and false discovery rate (FDR)–corrected *q*-values are presented.

## Results

### Participant characteristics

Participant characteristics are shown in Table 1. In total, 110 cognitively unimpaired individuals with an average age of 67.9 ± 5.7 years were included. The cohort included 50 monozygotic twin pairs. Of the 110 individuals, 41 (37.3%) carried an *APOE ɛ4* allele, and 8 (7.3%) were visually read as Aβ PET positive.^43^ Across both visits, all participants were classified as cognitively normal based on extensive neuropsychological testing.^41^ An overview of longitudinal neuropsychological test scores is provided in Supplementary Table 1.

**Table 1:** Demographic and clinical characteristics

	Total sample
*n*	110
Age, years	67.9 ± 5.7
Sex, female, *n* (%)	58 (53)
MMSE	
Baseline	29.1 ± 0.9
Follow-up	28.7 ± 1.3
Education level^a^	5.5 ± 1.0
APOE ε4 carrier, *n* (%)	41 (37)
Positive amyloid-PET (visual read BP_ND_ images), *n* (%)	8 (7)
Baseline	8 (7)
Follow-up	24 (22)
Global Aβ BP_ND_ (unscaled)	
Baseline	0.16 ± 0.11
Follow-up	0.18 ± 0.15

Shown are mean ± SD unless specified otherwise. All variables are derived from the baseline visit.

MMSE, mini mental state examination.

^a^Verhage education score (range 1–7, where higher scores indicate a higher education level^63^).

### Cross-sectional associations between early Aβ deposition and MEG measures

#### Early Aβ deposition is associated with slower oscillations and lower functional connectivity

**Figure 2:**
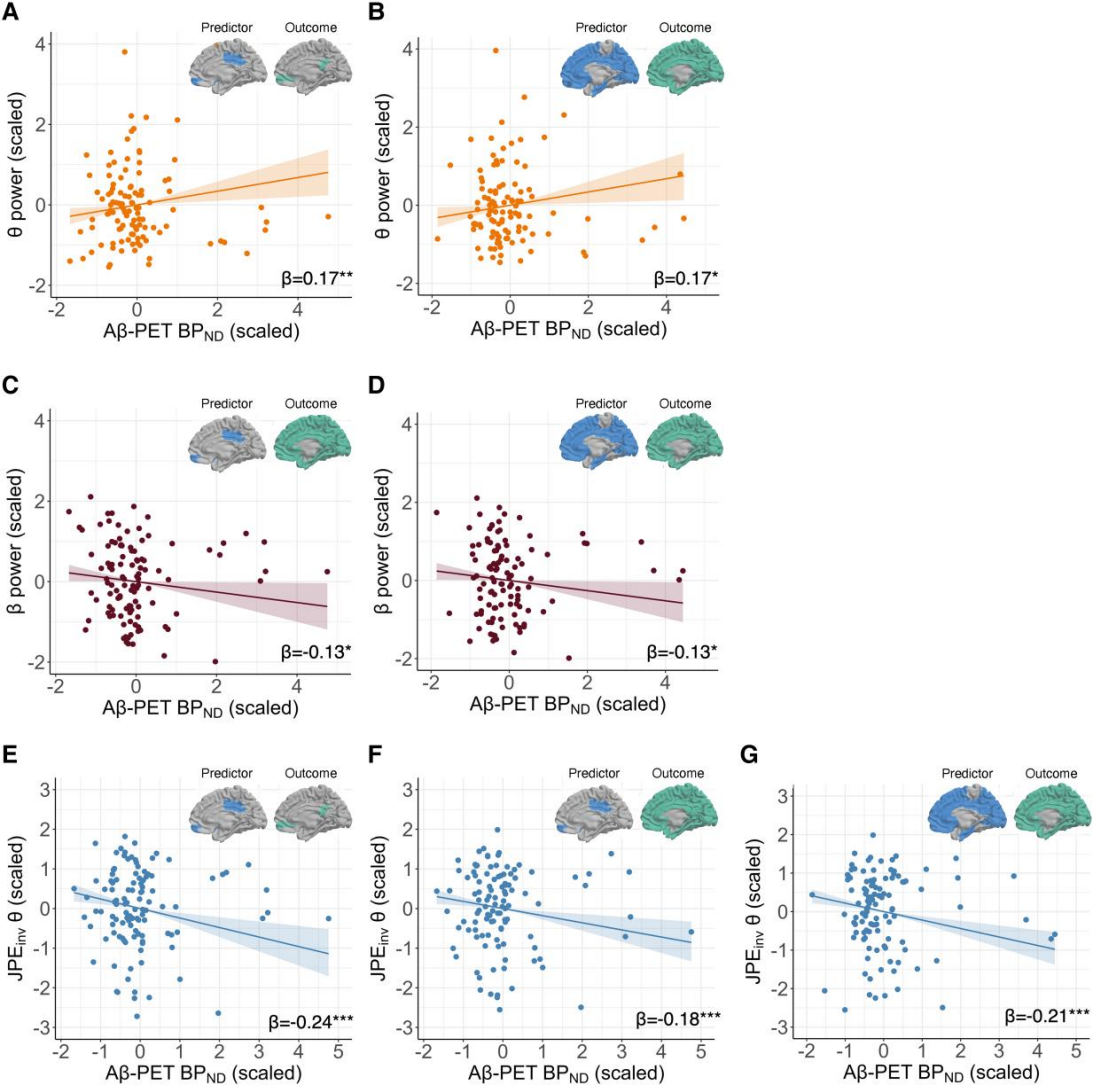
**Early Aβ deposition is associated with slower oscillations and reduced functional connectivity.** Displayed are baseline associations between *Z*-transformed Aβ BP_ND_ (*x*-axis) and *Z*-transformed MEG measures (*y*-axis). MEG measures were used as dependent variables in independent linear mixed models using Aβ BP_ND_ as independent variable and age and sex as covariates. Each data point represents an individual (*n* = 110). (**A**) Aβ BP_ND_ and theta power in the early Alzheimer’s disease ROI. (**B**) Whole-brain Aβ BP_ND_ and theta power. (**C**) Aβ BP_ND_ in the early Alzheimer’s disease ROI and whole-brain beta power. (**D**) Whole-brain Aβ BP_ND_ and beta power. (**E**) Aβ BP_ND_ in the early Alzheimer’s disease ROI and JPE_inv_ theta connectivity between the early Alzheimer’s disease ROI and the rest of the brain. (**F**) Aβ BP_ND_ in the early Alzheimer’s disease ROI and whole-brain JPE_inv_ theta connectivity. (**G**) Whole-brain Aβ BP_ND_ and JPE_inv_ theta connectivity. *P < 0.05; **P < 0.01; ***P < 0.001; Aβ, amyloid β; BP_ND_, non-displaceable binding potential.

We first tested cross-sectional associations between Aβ BP_ND_ and MEG measures (Fig. 2). Table 2 shows the LMM estimates and *P*-values for the different regions in which we investigated the relationship between Aβ BP_ND_ and MEG measures. Higher Aβ BP_ND_ in the early Alzheimer’s disease ROI, as well as across the whole brain, was associated with higher theta power in those same regions (Fig. 2A and B). Higher Aβ BP_ND_ in the early Alzheimer’s disease ROI and across the whole brain was associated with lower whole-brain beta power (Fig. 2C and D). A similar trend was observed for whole-brain Aβ BP_ND_ in relation to whole-brain alpha1 and gamma power: higher Aβ BP_ND_ tended to coincide with slower oscillations (i.e. positive trend with alpha1 power and negative trend with gamma power). Aβ BP_ND_ did not exhibit significant associations or trends with relative delta and alpha2 power.

**Table 2:** Cross-sectional associations between Aβ BP_ND_ and MEG measures

Outcome variable	Early Alzheimer’s disease ROI Aβ BP_ND_∼early Alzheimer’s disease ROI MEG	Early Alzheimer’s disease ROI Aβ BP_ND_∼whole-brain MEG	Whole-brain Aβ BP_ND_∼whole-brain MEG
**Oscillatory power**			
** **Delta power	−4.9e−04 (0.07)	0.01 (0.07)	−0.06 (0.06)
** **Theta power	0.17 (0.06)**/*	0.14 (0.07)#/n.s.	0.17 (0.07)*/*
** **Alpha1 power	0.09 (0.08)	0.09 (0.07)	0.12 (0.07)#
** **Alpha2 power	0.01 (0.07)	0.02 (0.08)	0.04 (0.07)
** **Beta power	−0.13 (0.07)#	−0.13 (0.06)*	−0.13 (0.06)*
** **Gamma power	−0.07 (0.08)	−0.12 (0.07)#	−0.12 (0.07)#
**Functional connectivity**			
** **JPE_inv_ theta	−0.24 (0.06)***/**	−0.18 (0.05)***/**	−0.21 (0.05)***/**
** **AEC-c alpha	−3.2e−03 (0.08)	−0.02 (0.08)	−0.01 (0.08)
** **PLI theta	−0.01 (0.08)	0.01 (0.08)	0.05 (0.08)

All LMM models are corrected for age and sex. Values are standardized beta (SE). We scaled predictor and outcome variables within each LMM to enable comparison of effect sizes. *P < 0.05; **P < 0.01; ***P < 0.001; #*P* < 0.1. For primary MEG measures, we report both uncorrected *P*-values and FDR-corrected *q*-values using the format */. Before the slash (/): uncorrected *P*-value. After the slash (/): FDR-corrected *q*-value. For example, **/* indicates a significance level of *P* < 0.01 and *Q* < 0.05. n.s., not significant.

Higher Aβ BP_ND_ in the early Alzheimer’s disease ROI was associated with lower JPE_inv_ theta connectivity of the early Alzheimer’s disease ROI with the rest of the brain (Fig. 2E) and across the whole brain (Fig. 2F). Elevated whole-brain Aβ BP_ND_ also showed a significant negative association with whole-brain JPE_inv_ theta connectivity (Fig. 2G). No significant cross-sectional associations were demonstrated between Aβ BP_ND_ and AEC-c alpha or PLI theta connectivity. While the associations between JPE_inv_ theta connectivity and Aβ BP_ND_ are only slightly stronger than those between the power measures and Aβ BP_ND_, they demonstrate markedly greater statistical significance. Overall, we report no clear differences between the effects observed in the early Alzheimer’s disease ROI, between the early Alzheimer’s disease ROI and the whole brain and across the whole brain (Table 2).

### Longitudinal associations between early Aβ deposition and MEG measures

#### Baseline Aβ deposition predicts oscillatory slowing and a reduction of functional connectivity over time

Next, we tested associations between baseline Aβ BP_ND_ and longitudinal MEG measures (Table 3). Higher Aβ BP_ND_ in the early Alzheimer’s disease ROI at baseline was associated with faster oscillatory slowing over time, reflected by a steeper increase in theta power over time in the early Alzheimer’s disease ROI and across the whole brain, as well as a decrease in whole-brain beta power over time. Higher baseline whole-brain Aβ BP_ND_ was associated with a steeper increase in whole-brain theta power over time. Notably, higher Aβ BP_ND_ in the early Alzheimer’s disease ROI at baseline was associated with a steeper decrease in delta power over time in the early Alzheimer’s disease ROI. Finally, we report an association between higher Aβ BP_ND_ in the early Alzheimer’s disease ROI at baseline and a decrease in JPE_inv_ theta connectivity over time between the early Alzheimer’s disease ROI and the rest of the brain. We report no significant longitudinal associations for the remaining measures (alpha1, alpha2 and gamma power, AEC-c alpha and PLI theta connectivity).

**Table 3:** Associations between baseline Aβ BP_ND_ and longitudinal MEG measures

Outcome variable	Early Alzheimer’s disease ROI Aβ BP_ND_∼early Alzheimer’s disease ROI MEG	Early Alzheimer’s disease ROI Aβ BP_ND_∼whole-brain MEG	Whole-brain Aβ BP_ND_∼whole-brain MEG
**Oscillatory power**			
** **Delta power	−0.08 (0.03)*	−0.03 (0.02)	−0.03 (0.02)
** **Theta power	0.11 (0.03)***/***	0.08 (0.02)***/***	0.08 (0.02)**/**
** **Alpha1 power	0.03 (0.02)	0.02 (0.02)	0.02 (0.02)
** **Alpha2 power	0.05 (0.03)	0.01 (0.02)	2.6e−03 (0.02)
** **Beta power	−0.01 (0.03)	−0.04 (0.02)*	−0.04 (0.02)#
** **Gamma power	−0.02 (0.04)	−0.02 (0.02)	−0.02 (0.02)
**Functional connectivity**			
** **JPE_inv_ theta	−0.07 (0.03)*/n.s.	−0.04 (0.02)	−0.03 (0.02)
** **AEC-c alpha	0.01 (0.04)	0.01 (0.03)	0.01 (0.03)
** **PLI theta	0.06 (0.05)	0.05 (0.04)	0.03 (0.04)

All LMM models are corrected for age and sex. Values are standardized beta (SE). We scaled predictor and outcome variables within each LMM to enable comparison of effect sizes. *P < 0.05; **P < 0.01; ***P < 0.001; #*P* < 0.1. For primary MEG measures, we report both uncorrected *P*-values and FDR-corrected *q*-values using the format */. Before the slash (/): uncorrected *P*-value. After the slash (/): FDR-corrected *q*-value. For example, **/* indicates a significance level of *P* < 0.01 and *Q* < 0.05.

n.s., not significant.

#### Baseline MEG measures do not predict longitudinal Aβ deposition

We also investigated whether baseline MEG measures were associated with the level of Aβ deposition at baseline and over time. These analyses did not yield any statistically significant findings (Supplementary Tables 2 and 3).

#### Longitudinal changes in Aβ deposition are associated with longitudinal changes in oscillatory power and functional connectivity

**Figure 3:**
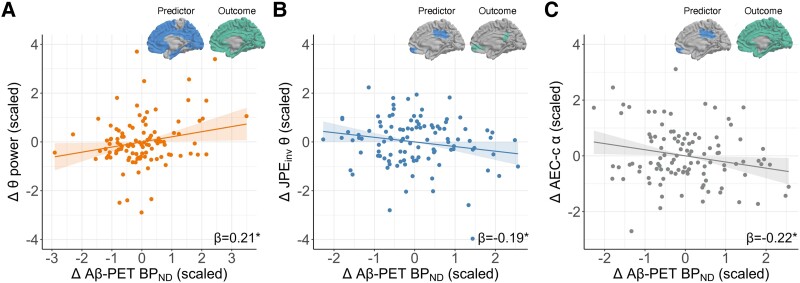
**Change in Aβ deposition is associated with changes in oscillatory power and functional connectivity.** Displayed are associations between *Z*-transformed annual changes in Aβ BP_ND_ (*x*-axis) and *Z*-transformed annual changes in MEG measures (*y*-axis). Changes in MEG measures were used as dependent variables in independent linear mixed models using change in Aβ BP_ND_ as independent variable and age and sex as covariates. Each data point represents an individual (*n* = 107). (**A**) Annual change in whole-brain Aβ BP_ND_ and theta power. (**B**) Annual change in Aβ BP_ND_ in the early Alzheimer’s disease ROI and JPE_inv_ theta connectivity between the early Alzheimer’s disease ROI and the rest of the brain. (**C**) Annual change in Aβ BP_ND_ in the early Alzheimer’s disease ROI and whole-brain AEC-c alpha connectivity. Δ Aβ PET BP_ND_ was missing for *n* = 2. *n* = 1 outlier was removed. *Δ*, annual change; *P < 0.05; Aβ, amyloid β; BP_ND_, non-displaceable binding potential.

To investigate whether longitudinal changes in Aβ deposition are associated with changes in neuronal functioning over time, we examined the relationship between the annual change in Aβ BP_ND_ and MEG measures (Table 4). Longitudinal changes in whole-brain Aβ BP_ND_ were positively associated with longitudinal changes in whole-brain theta power (Fig. 3A). Changes in Aβ BP_ND_ in the early Alzheimer’s disease ROI were negatively associated with changes in JPE_inv_ theta connectivity between the early Alzheimer’s disease ROI and the rest of the brain (Fig. 3B) as well as whole-brain AEC-c alpha connectivity (Fig. 3C) over time.

**Table 4:** Associations between the annual change in Aβ BP_ND_ and the annual change in MEG measures

Outcome variable	Δ Early Alzheimer’s disease ROI Aβ BP_ND_∼Δ early Alzheimer’s disease ROI MEG	Δ Early Alzheimer’s disease ROI Aβ BP_ND_∼Δ whole-brain MEG	Δ Whole-brain Aβ BP_ND_∼Δ whole-brain MEG
**Oscillatory power**			
** **Delta power	0.02 (0.10)	0.04 (0.10)	−0.01 (0.10)
** **Theta power	0.07 (0.10)	0.09 (0.10)	0.21 (0.10)*/n.s.
** **Alpha1 power	−0.13 (0.10)	−0.05 (0.10)	0.02 (0.10)
** **Alpha2 power	0.05 (0.10)	0.07 (0.10)	0.08 (0.10)
** **Beta power	0.02 (0.10)	−0.08 (0.10)	−0.14 (0.10)
** **Gamma power	−0.05 (0.10)	−0.04 (0.10)	−0.11 (0.10)
**Functional connectivity**			
** **JPE_inv_ theta	−0.19 (0.09)*/n.s.	−0.09 (0.10)	−0.13 (0.10)
** **AEC-c alpha	−0.14 (0.09)	−0.22 (0.09)*	−0.11 (0.10)
** **PLI theta	−0.11 (0.10)	−3.6e−03 (0.10)	0.11 (0.10)

All LMM models are corrected for age and sex. Values are standardized beta (SE). Δ Aβ PET BP_ND_ was missing for *n* = 2. *n* = 1 outlier was removed. We scaled predictor and outcome variables within each LMM to enable comparison of effect sizes. *Δ*, annual change; *P < 0.05. For primary MEG measures, we report both uncorrected *P*-values and FDR-corrected *q*-values using the format */. Before the slash (/): uncorrected *P*-value. After the slash (/): FDR-corrected *q*-value. For example, **/* indicates a significance level of *P* < 0.01 and *Q* < 0.05.

n.s., not significant.

One participant, exhibiting notably greater annual change in Aβ BP_ND_ in both the early Alzheimer’s disease ROI (*Z*-transformed value = 6.1) and across the whole brain (*Z*-transformed value = 7.1) compared with all other participants, was excluded from this analysis to prevent excessive influence on the results. Results incorporating this participant can be found in Supplementary Table 4. Significant outcomes are visualized in Supplementary Fig. 1. The observed trends remained consistent: change in Aβ BP_ND_ was positively associated with an increase in oscillatory slowing and a reduction in AEC-c alpha and JPE_inv_ theta connectivity over time. The magnitudes of these effects were considerably larger when this participant was included. In addition, we report a significant positive association between changes in whole-brain Aβ BP_ND_ and PLI theta connectivity over time.

## Discussion

Aberrations in neuronal activity and their link to Aβ pathology have been extensively explored in animal models of Alzheimer’s disease.^3-7^ More recent studies have begun to unravel the functional alterations related to Aβ deposition in patients on the Alzheimer’s disease continuum.^34-37^ In this longitudinal MEG-PET study, we showed that Aβ deposition is associated with slower oscillations and lower functional connectivity in cognitively unimpaired individuals. Additionally, we found that baseline Aβ deposition is associated with future oscillatory slowing and a reduction of functional connectivity, while baseline MEG measures were not associated with future Aβ deposition. Finally, we showed that longitudinal changes in Aβ deposition are associated with changes in MEG measures over time. These data suggest that MEG is a sensitive method for detecting neuronal dysfunction related to Aβ deposition in cognitively unimpaired individuals.

### Early Aβ deposition is associated with slower oscillations and lower functional connectivity

Aβ deposition in orbitofrontal and posterior cingulate regions (i.e. early Alzheimer’s disease regions) and across the whole brain was associated with increased theta power in those same regions, as well as decreased whole-brain beta power. It is important to note that throughout the study, only results for primary MEG measures of interest (theta power and functional connectivity measured by JPE_inv_ theta) were corrected for multiple comparisons. Results for secondary MEG measures should therefore be interpreted with caution. Our findings align with prior research indicating an association between Aβ pathology and oscillatory slowing in individuals with prodromal and probable Alzheimer’s disease^35^ and suggest that the observed relationship represents a continuum from pre-clinical Alzheimer’s disease to dementia. Oscillatory slowing is an early neurophysiological hallmark of Alzheimer’s disease, which has been observed as early as the subjective cognitive decline stage of the disease.^61^ Increased relative theta power has moreover been reported in cognitively unimpaired carriers of APP and/or PSEN1 mutations.^64^ In contrast to these findings, Nakamura *et al*.^37^ reported significantly higher pre-frontal alpha power in Aβ-positive cognitively unimpaired individuals and patients with prodromal Alzheimer’s disease compared with Aβ-negative individuals. More recently, a study demonstrated that Aβ co-localizes with accelerated neurophysiological activity—characterized by increased alpha and decreased delta power—in cognitively unimpaired individuals.^65^ This effect was reduced in individuals with greater tau pathology. The latter two studies support the hypothesis of a potential non-linear relationship between Aβ and oscillatory dynamics: in early pre-clinical stages, amyloid-induced hyper-activity may lead to increased fast activity, while in later stages, tau-related hypoactivity could result in increased slow activity. The findings of the present study do not align with this compelling hypothesis but rather support the idea of progressive oscillatory slowing over time. Through computational modelling, we have attempted to explain our findings, and we have demonstrated that the slowing of large-scale oscillations that is observed in patients on the Alzheimer’s disease continuum is compatible with both hyper-activity in early stages of the disease and late-stage neuronal hypoactivity.^66,67^ Two important differences between the present study and the study by Gallego-Rudolf *et al*.^65^ should be noted. First, we did not examine the potential impact of tau burden on the observed associations, which may have influenced our results. Second, Gallego-Rudolf *et al*.^65^ analysed eyes-open MEG data, whereas our analysis focused on eyes-closed data. Opening of the eyes leads to suppression of the posterior dominant alpha rhythm, altering the results of spectral analyses significantly.^68,69^ The extent and implications of this difference on the reported findings remain unclear.

We identified negative associations between Aβ deposition and JPE_inv_ theta connectivity between early Alzheimer’s disease regions and the rest of the brain, as well as whole-brain JPE_inv_ theta connectivity. This is in line with previous work, in which we observed a widespread decrease in JPE_inv_ theta connectivity in patients with Aβ-positive prodromal Alzheimer’s disease compared with Aβ-negative controls.^33^ The present study reports no cross-sectional associations between Aβ deposition and AEC-c alpha or PLI theta connectivity. In early Alzheimer’s disease, toxic effects of Aβ are known to contribute to neuronal hyper-excitability and activity.^70-72^ The neuronal excitation–inhibition (E–I) balance, which reflects the inter-play between excitatory and inhibitory neuronal activity, is disrupted. The E–I balance can shift rapidly due to changes in neuronal firing patterns, synaptic input or network dynamics. The JPE_inv_ might be more effective at detecting these rapid fluctuations in neuronal activity and connectivity than traditional measures, due to its high temporal resolution. Specifically, the JPE_inv_ captures faster changes in the signal by evaluating connectivity on shorter timescales, while measures like phase or amplitude envelope correlations are computed over longer time periods. Two previous computational modelling studies demonstrated that the JPE_inv_ is indeed sensitive to changes in E–I balance^73^ and that it outperforms AEC-c and PLI in detecting network hyper-excitability in early Alzheimer’s disease.^74^ This could account for the stronger associations observed between JPE_inv_ and Aβ deposition compared with those found with AEC-c and PLI.

Phase locking or synchronization of neuronal activity is crucial for effective communication between groups of neurons.^75^ Aβ-induced neuronal hyper-activity in pre-clinical Alzheimer’s disease may influence the ability of neurons to precisely coordinate their activity, for instance by increasing background noise or impairing inhibitory control, theoretically resulting in a reduction of functional connectivity, as reported in this study. Both increased and decreased functional connectivities have previously however been related to Aβ pathology in cognitively unimpaired individuals.^36,76-78^ One explanation for the discrepant reports could be that both hyper- and hypo-connectivity effects are at play on different time scales. The size of the neuronal populations engaged in synchronized activity affects the frequency of oscillations, considering that most neuronal connections are local. Oscillations at higher frequencies typically involve a smaller group of neurons that are tightly connected to each other, while slow oscillations engage widespread neuronal populations, allowing for the coordination of activity over larger brain areas.^79^ This relationship between anatomical structure and oscillatory patterns enables the brain to operate at multiple temporal and spatial scales simultaneously. Impairments in the temporal coordination of neuronal activity due to Aβ pathology may result in hyper-connectivity on one temporal scale (e.g. in the theta band), but hypo-connectivity on another (e.g. in the alpha band). Second, the choice of functional connectivity metric can influence whether an increase or decrease of connectivity is observed. Different metrics focus on different aspects of oscillations, such as amplitude or phase, thereby capturing distinct aspects of neuronal dynamics.^80^ These factors highlight the complexity of a direct comparison of functional connectivity findings across studies that employ different metrics or modalities that operate on varying time scales (e.g. MEG and fMRI).

Unexpectedly, the observed associations were not stronger in early Alzheimer’s disease regions than across the whole brain. Neuronal activity and synchronization are less constrained by physical boundaries than Aβ deposition. As a result, functional disruption caused by Aβ pathology may manifest more diffusely across the brain, with widespread effects on neuronal circuits. This could potentially account for the observed lack of regional specificity in our findings.

### Baseline Aβ predicts oscillatory slowing and reduction of functional connectivity over time

Aβ deposition in early Alzheimer’s disease regions at baseline was associated with accelerated oscillatory slowing over time, reflected by an increase in theta power and decrease in beta power across the whole brain. Baseline whole-brain Aβ deposition was associated with an increase in whole-brain theta power over time. The opposite was not true: baseline MEG measures were not associated with future Aβ deposition. Aβ-dependent neuronal hyper-activity is described as a vicious cycle: Aβ oligomers obstruct glutamate reuptake, leading to peri-synaptic glutamate accumulation and neuronal depolarization, thereby promoting hyper-activity.^12^ This hyper-activity, in turn, can stimulate further Aβ oligomer production, perpetuating the cycle and advancing the disease.^4,8,11^ It is conceivable that MEG measures are more sensitive to changes in soluble Aβ oligomer concentrations—undetectable using PET—than the formation of plaques, potentially explaining their limited predictive capability. The modest alterations in Aβ deposition over time within our cohort may furthermore have complicated accurate predictions of change. Therefore, while the results of this study suggest that Aβ deposition predicts neuronal dysfunction rather than the reverse, we cannot conclude that this process is unidirectional. Different results may be observed with longer follow-up periods, techniques that are more sensitive to other forms of Aβ, or in participants exhibiting larger changes in Aβ deposition over time.

Baseline Aβ deposition in early Alzheimer’s disease regions was associated with a decrease in delta power over time within those same regions. This association diverges from the broader observed trend, which suggests that Aβ deposition is linked to oscillatory slowing. To better understand this finding, we examined the distribution of delta power across the whole brain, averaged across all participants, at baseline and follow-up (Supplementary Fig. 3). Notably, the orbitofrontal gyri, included in the early Alzheimer’s disease regions, exhibit the highest levels of delta power. This is commonly attributed to eye-blink artefacts in M/EEG recordings.^81^ Despite rigorous artefact rejection efforts, the effect persisted in the analysed data, likely introducing some degree of uncertainty into the delta band results. To address this, we conducted a *post hoc* analysis, in which we assessed the association between baseline Aβ deposition in the early Alzheimer’s disease regions and delta power in only the posterior cingulate cortices over time. As indicated in Supplementary Table 5, the previously observed interaction ceased to be significant. As this reported result may be influenced in part by the presence of ocular artefacts in the data, it should be interpreted with caution.

### Change in Aβ deposition is associated with changes in oscillatory power and functional connectivity

We found a positive association between longitudinal changes in whole-brain Aβ deposition and whole-brain theta power. Moreover, longitudinal changes in Aβ deposition in early Alzheimer’s disease regions were negatively associated with changes in whole-brain AEC-c alpha connectivity and JPE_inv_ theta connectivity between early Alzheimer’s disease regions and the rest of the brain. These findings provide insight into how Aβ pathology and neurophysiological measures evolve relative to each other.

### Strengths and limitations

Strengths of the study include the large, well-characterized study population and the use of longitudinal Aβ PET and source-level MEG data. The study also has some limitations. First, the cohort consisted of a large percentage of Aβ negative individuals (as based on visual read of the [^18^F]-flutemetamol PET images according to GE HealthCare guidelines^82^), resulting in relatively low overall levels of Aβ deposition at baseline and over time. Analysing Aβ as a continuous variable across the full cohort, rather than dichotomizing participants into Aβ-positive and Aβ-negative subgroups, allowed us to examine the full spectrum of Aβ-related neurophysiological changes, recognizing that even low or subthreshold Aβ levels may have important implications for brain function. While it is possible that some of the observed associations may reflect healthy ageing processes rather than early Alzheimer’s disease pathology, this approach provides a more comprehensive understanding of the relationship between Aβ pathology and MEG measures. Defining a strict threshold for positivity overlooks the nuances in the progression of amyloid accumulation and may miss important patterns that lie between the extremes. Supplementary Fig. 2 visualizes the (unscaled) longitudinal change in Aβ deposition between baseline and follow-up for each participant, both on whole-brain level and within the early Alzheimer’s disease ROI. Participants in the Aβ-positive range, as well as those who converted to Aβ-positivity during follow-up, show a largely consistent increase in Aβ deposition over time, indicating a meaningful accumulation of Aβ. Participants in the Aβ-negative range display more variability, with both increases and decreases in Aβ deposition over time. This noise—an inherent challenge when studying cognitively unimpaired individuals with low Aβ levels—should be taken into consideration when interpreting the results. One participant displaying a substantial increase in Aβ deposition over time was excluded from the main analysis. Inclusion of this participant resulted in more pronounced associations between longitudinal changes in Aβ deposition and MEG measures (Supplementary Table 4 and  Fig. 1). These findings suggest that incorporating a larger group of cognitively unimpaired individuals with elevated Aβ deposition (or a larger change in Aβ deposition over time) may enhance the strength of the observed associations. A potential explanation for the low proportion of Aβ-positive participants in this study could be that follow-up visits took place during the COVID-19 pandemic. The follow-up protocol of the EMIF-AD PreclinAD study is extensive, involving neuropsychological testing, blood collection, CSF collection, MRI, PET, optical coherence tomography and MEG.^41^ Some participants were reluctant to attend the hospital during the pandemic, leading to missed follow-up visits or shortened protocols. This may have disproportionately affected Aβ-positive individuals, who might have been more hesitant to come in for follow-up assessments, resulting in a smaller proportion of Aβ-positive participants with longitudinal MEG recordings and PET scans available. Next, we made use of distinct atlases for the Aβ PET and MEG analysis. A probabilistic atlas, such as the Hammers atlas, can help mitigate partial volume effects, enabling more accurate quantification of PET signals.^46^ Since the need for a probabilistic atlas is less pronounced in MEG analysis, we opted for a brain atlas with higher spatial resolution (i.e. the AAL atlas). While the early Alzheimer’s disease regions from the two atlases do not entirely align, this discrepancy does not undermine the significance or direction of the reported associations. This study only focused on periodic properties of the MEG signals. Recent research largely indicates that periodic, rather than aperiodic, neurophysiology is associated with Alzheimer’s disease,^83-86^ although there are some counterpoints.^87,88^ It remains unclear whether the effects reported in this study are confounded by aperiodic shifts. Moreover, while we leveraged a unique, well-characterized cohort of cognitively unimpaired older adults, our study sample was predominantly white and highly educated. Our findings might therefore not extrapolate to the entire population. Future studies should aim to include a more diverse sample with varying educational backgrounds to address this potential confound. Finally, the present study investigated whether MEG measures could serve as early indicators of Aβ-dependent neuronal hyper-activity without taking other disease factors that are more closely related to clinical progression, such as APOE status and tau burden, into consideration. Future research should explore the influence of risk factors of cognitive decline on the reported associations.

## Conclusion

Disruptions of oscillatory power and functional connectivity appear to represent early functional consequences of emerging Aβ pathology in cognitively unimpaired individuals. Not only baseline Aβ levels but also temporal changes in Aβ levels inform us about the trajectory of neurophysiological characteristics over time. MEG measures may therefore hold promise as biomarkers for monitoring disease progression and evaluating the effects of Aβ-targeted therapies on neuronal activity in pre-clinical Alzheimer’s disease.

## Supplementary Material

fcaf018_Supplementary_Data

## Data Availability

The data that support the findings of this study are available from the corresponding author, upon reasonable request.
