# Supplementary material for: Amyloid-β deposition predicts oscillatory slowing of magnetoencephalography signals and a reduction of functional connectivity over time in cognitively unimpaired adults
Source: Brain Commun. 2025 Jan 20;7(1):fcaf018. doi: 10.1093/braincomms/fcaf018 (PMC11851009; doi:10.1093/braincomms/fcaf018)
Supplement: fcaf018_Supplementary_Data [file fcaf018_supplementary_data.docx]

**Supplementary material**

**Supplementary methods -** Details of the linear mixed effect model analyses

Linear mixed effects models (LMMs) were performed separately and independently for each MEG outcome measure (N = 9) and level of regional analysis (N = 3). We explain the univariate LMMs with example pseudocode for R (version 4.2.1, lme4 package).

1. **Investigating the association between baseline Aβ BP_ND_ and MEG measures at baseline and over time.**

Model A1 (without interaction)

**Formula:**

MEG_longitudinal ~ Time_years + Aβ_baseline + (1|Family_ID) + (1|Family_ID:Individual_ID) + age + sex

**Interpretation:**

This model examines how baseline Aβ BP_ND_ (Aβ_baseline) and time (Time_years) independently affect longitudinal MEG measures (MEG_longitudinal), controlling for age and sex. The model accounts for random effects from family grouping (Family_ID) and individuals nested within families (FamilyID:Individual_ID).

**Key terms:**

- Time_years: The number of years (continuous) between baseline and follow-up visits for each participant.
- Aβ_baseline: Baseline Aβ BP_ND._
- (1|Family_ID) and (1|Family_ID:Individual_ID): Random effects to account for the fact that individuals are related (family structure) and that measurements may vary across individuals within a family.

Model A2 (with interaction)

**Formula:**

MEG_longitudinal ~ Time_years + Aβ_baseline + Time_years:Aβ_baseline + (1|Family_ID) + (1|FamilyID:Individual_ID) + age + sex

**Interpretation:**

This model introduces an interaction between Time_years and Aβ_baseline to determine if the association between baseline Aβ BP_ND_ and MEG measures changes over time. If the interaction is significant, it indicates that the effect of baseline Aβ BP_ND_ on MEG measures is not constant but changes over time.

**Key terms:**

- **Time_years:A**β**_baseline:** This interaction term tests whether the relationship between baseline Aβ BP_ND_ and MEG measures changes over time.

We did not run separate models without a time variable to estimate baseline associations. This approach allowed us to leverage all available data (increasing statistical power), control for potential confounding effects of time and ensure consistency across cross-sectional and longitudinal analyses. If the interaction term in Model A2 (Time_years:Aβ_baseline) was not significant, the cross-sectional association was estimated using the simpler Model A1. However, if the interaction term in Model A2 was significant, indicating that the relationship between baseline Aβ BP_ND_ and MEG measures changes over time, the cross-sectional association was derived from Model A2. For the association between baseline Aβ BP_ND_ and MEG measures over time, the interaction effect (Time_years:Aβ_baseline) from Model A2 was reported.

1. **Investigating the association between baseline MEG measures and Aβ BP_ND_ both at baseline and over time.**

Model B1 (without interaction)

**Formula:**

Aβ_longitudinal ~ Time_years + MEG_baseline + (1|Family_ID) + (1|Family_ID:Individual_ID) + age + sex

**Interpretation:**

This model examines how baseline MEG measures (MEG_baseline) and time (Time_years) independently affect longitudinal Aβ BP_ND_ (Aβ_longitudinal), controlling for age and sex. The model accounts for random effects from family grouping (Family_ID) and individuals nested within families (FamilyID:Individual_ID).

**Key terms:**

- Time_years: The number of years (continuous) between baseline and follow-up visits for each participant.
- MEG_baseline: Baseline MEG measure.
- (1|Family_ID) and (1|Family_ID:Individual_ID): Random effects to account for the fact that individuals are related (family structure) and that measurements may vary across individuals within a family.

Model B2 (with interaction)

**Formula:**

Aβ_longitudinal ~ Time_years + MEG_baseline + Time_years:MEG_baseline + (1|Family_ID) + (1|FamilyID:Individual_ID) + age + sex

**Interpretation:**

This model introduces an interaction between Time_years and MEG_baseline to determine if the association between baseline MEG measures and Aβ BP_ND_ changes over time. If the interaction is significant, it indicates that the effect of baseline MEG measures on Aβ BP_ND_ is not constant but changes over time.

**Key terms:**

- **Time_years:MEG_baseline:** This interaction term tests whether the relationship between baseline MEG measures and Aβ BP_ND_ changes over time.

We did not run separate models without a time variable to estimate baseline associations. This approach allowed us to leverage all available data (increasing statistical power), control for potential confounding effects of time and ensure consistency across cross-sectional and longitudinal analyses. If the interaction term in Model B2 (Time_years:MEG_baseline) was not significant, the cross-sectional association was estimated using the simpler Model B1. However, if the interaction term in Model B2 was significant, indicating that the relationship between baseline MEG measures and Aβ BP_ND_ changes over time, the cross-sectional association was derived from Model B2. For the association between baseline MEG measures and Aβ BP_ND_ over time, the interaction effect (Time_years:MEG_baseline) from Model B2 was reported.

1. **Investigating the association between change in Aβ BP_ND_ and change in MEG measures over time.**

Model C

**Formula:**

Yearly_change_MEG ~ Yearly_change_Aβ + (1|Family_ID) + age + sex

**Interpretation:**

This model examines how the yearly change in MEG measures is associated with the yearly change in Aβ BP_ND_. Specifically, it evaluates whether variations in the rate of change in Aβ BP_ND_ are related to variations in the rate of change in MEG measures over time. Additionally, it controls for the effects of age and sex and accounts for random variability due to familial relationships.

**Key Terms:**

- Yearly_change_MEG: The rate of change in MEG measures per year.
- Yearly_change_Aβ: The rate of change in Aβ BP_ND_ per year.
- (1|Family_ID): A random effect term that accounts for the correlation of MEG and Aβ BP_ND_ changes within families. Note that Model C does not include a random effect for individuals, as only one change score is available per participant.

To describe the association between changes in Aβ BP_ND_ and MEG measures over time, we report the estimate for the independent variable of interest, Yearly_change_Aβ.

**Supplementary Table 1** Neuropsychological test scores at baseline and follow-up

| Neuropsychological Test | Range | Participants completed BL | Mean (SD) or Median [IQR] BL | Participants completed FUP | Mean (SD) or Median [IQR] FUP |
| --- | --- | --- | --- | --- | --- |
| MMSE | 0-30 | 110 | 29.1(0.9) | 110 | 28.7 (1.3) |
| RAVLT |  | 110 |  | 110 |  |
| - Immediate recall | 0-75 |  | 44.1 (8.7) |  | 43.6 (13.2) |
| - Delayed recall 20 minutes | 0-15 |  | 8.5 (2.8) |  | 8.8 (3.3) |
| - Learning |  |  | 6.0 (1.8) |  | 5.8 (2.3) |
| - Recognition | 0-30 |  | 28.5 (2.0) |  | 28.5 (1.9) |
| WAIS-III digit span |  | 110 |  | 110 |  |
| - Forward Score | 0-14 |  | 8.9 (1.9) |  | 8.8 (1.7) |
| - Forward Score Digit Span | 0-8 |  | 6.0 (1.1) |  | 5.9 (1.0) |
| - Backwards Score | 0-14 |  | 6.0 (1.5) |  | 6.2 (1.6) |
| - Backwards Score Digit Span | 0-8 |  | 4.5 (1.0) |  | 4.6 (1.1) |
| FNAME |  | 104 |  | 109 |  |
| - Total names | 0-48 |  | 20.6 (9.6) |  | 23.2 (11.1) |
| - Total occupation | 0-48 |  | 33.8 (8.4) |  | 33.4 (8.8) |
| - Total names and occupation | 0-96 |  | 54.4 (16.8) |  | 56.5 (19.0) |
| Rey Complex Figure Test |  | 110 |  | 110 |  |
| - Copy |  |  | 34.0 (2.3) |  | 33.6 (2.3) |
| - Delayed copy 3min |  |  | 19.4 (5.3) |  | 20.5 (5.9) |
| - Delayed copy 20min |  |  | 19.2 (4.9) |  | 19.1 (12.7) |
| TMT A & B |  | 110 |  | 110 |  |
| - TMT A | seconds |  | 36.0 [29-44] |  | 34.0 [28-43] |
| - TMT A errors |  |  | 0 [0-0] |  | 0 [0-0] |
| - TMT B | seconds |  | 84.5 [67-110] |  | 88.0 [67-107] |
| - TMT B errors |  |  | 0 [0-1] |  | 0 [0-1] |
| Graded naming | 0-30 | 110 | 18.6 (3.3) | 110 | 19.1 (3.4) |
| CANTAB |  |  |  | 0 |  |
| - RVP-A |  | 102 | 0.86 (0.14) |  |  |
| - RVP Median response latency | miliseconds | 103 | 452.7 (95.9) |  |  |
| - PAL Total errors adjusted |  | 110 | 27.1 (16.1) |  |  |
| - RTI Simple median RT |  | 110 | 296.0 (55.2) |  |  |
| - RTI Five choices median RT |  | 110 | 326.9 (42.2) |  |  |
| - RTI SD five choices RT |  | 100 | 59.0 (26.9) |  |  |
| - SWM Between errors |  | 109 | 19.0 (8.9) |  |  |
| - SWM Strategy |  | 109 | 17.9(2.7) |  |  |
| Verbal Fluency |  | 110 |  | 110 |  |
| - In Dutch: letters D, A and T |  |  | 38.6 (10.7) |  | 39.2 (10.4) |
| - Category fluency animal 1 minute |  |  | 22.9 (7.0) |  | 23.7 (4.9) |
| - Category fluency animal 2 minutes |  |  | 35.6 (10.0) |  | 36.2 (7.9) |
| VAT |  | 110 |  | 110 |  |
| - A 2 trials | 0-12 |  | 12 [12-12] |  | 12 [12-12] |
| - B 2 trials | 0-12 |  | 12 [11-12] |  | 11 [11-12] |
| - Naming | 0-12 |  | 12 [12-12] |  | 12 [12-12] |

*MMSE: Mini Mental State Examination, RAVLT: Rey auditory verbal learning test, WAIS: Wechsler Adult Intelligence Scale, F-NAME: Face-name associative memory exam TMT: Trail Making Test, CANTAB: Cambridge Neuropsychological Test Automated Battery, VAT: Visual Association Test.*

**Supplementary Table 2** Cross-sectional associations between MEG measures and amyloid-β BP_ND_

| Outcome variable | Early AD ROI MEG ~ Early AD ROI Aβ BP_ND_ | Whole-brain MEG ~ Early AD ROI Aβ BP_ND_ | Whole-brain MEG ~  Whole-brain Aβ BP_ND_ |
| --- | --- | --- | --- |
| Oscillatory power |  |  |  |
| Delta power | 0.07 (0.09) | -4.7E-03 (0.10) | -0.04 (0.10) |
| Theta power | -0.04 (0.09) | 0.01 (0.09) | 0.03 (0.10) |
| Alpha1 power | 0.06 (0.09) | 0.10 (0.10) | 0.08 (0.10) |
| Alpha2 power | 0.04 (0.09) | 0.10 (0.09) | 0.11 (0.10) |
| Beta power | -0.08 (0.09) | -0.07 (0.10) | -0.06 (0.10) |
| Gamma power | -0.04 (0.08) | -0.12 (0.09) | -0.10 (0.10) |
|  |  |  |  |
| Functional connectivity |  |  |  |
| JPE_inv_ theta | 0.03 (0.10) | -0.04 (0.10) | -0.07 (0.11) |
| AEC-c alpha | 0.11 (0.09) | 0.13 (0.09) | 0.12 (0.09) |
| PLI theta | -0.07 (0.08) | -0.06 (0.09) | -0.06 (0.09) |

All LMM models are corrected for age and sex. Values are standardized beta (SE). Follow-up Aβ-PET BP_ND_ was missing for *n* = 2. We scaled predictor and outcome variables within each LMM to enable comparison of effect sizes.

| Outcome variable | Early AD ROI MEG ~ Early AD ROI Aβ BP_ND_ | Whole-brain MEG ~ Early AD ROI Aβ BP_ND_ | Whole-brain MEG ~  Whole-brain Aβ BP_ND_ |
| --- | --- | --- | --- |
| Oscillatory power |  |  |  |
| Delta power | 0.01 (0.04) | 0.01 (0.04) | 0.01 (0.03) |
| Theta power | 0.01 (0.04) | 0.04 (0.04) | 0.05 (0.03) |
| Alpha1 power | 0.01 (0.04) | 0.02 (0.04) | -0.004 (0.03) |
| Alpha2 power | -0.01 (0.04) | -0.02 (0.04) | 0.002 (0.03) |
| Beta power | -0.01 (0.04) | -0.04 (0.04) | -0.02 (0.03) |
| Gamma power | -0.01 (0.04) | -0.03 (0.04) | -0.03 (0.03) |
|  |  |  |  |
| Functional connectivity |  |  |  |
| JPE_inv_ theta | 0.01 (0.04) | -0.02 (0.04) | -0.02 (0.03) |
| AEC-c alpha | 0.03 (0.04) | 0.05 (0.04) | 0.05 (0.03) |
| PLI theta | 0.01 (0.04) | 0.02 (0.04) | 0.01 (0.03) |

**Supplementary Table 3** Associations between baseline MEG measures and longitudinal amyloid-β BP_ND_

All LMM models are corrected for age and sex. Values are standardized beta (SE). Follow-up Aβ-PET BP_ND_ was missing for *n* = 2. We scaled predictor and outcome variables within each LMM to enable comparison of effect sizes.

**Supplementary Table 4** Associations between the annual change in amyloid-β BP_ND_ and the annual change in MEG measures (including outlier)

| Outcome variable | Δ Early AD ROI Aβ BP_ND_ ~ Δ Early AD ROI MEG | Δ Early AD ROI Aβ BP_ND_ ~ Δ Whole-brain MEG | Δ Whole-brain Aβ BP_ND_ ~ Δ Whole-brain MEG |
| --- | --- | --- | --- |
| Oscillatory power |  |  |  |
| Delta power | -0.03 (0.09) | -0.08 (0.10) | -0.14 (0.10) |
| Theta power | 0.24 (0.10)* | 0.16 (0.10)# | 0.26 (0.10)** |
| Alpha1 power | -0.06 (0.10) | -0.03 (0.10) | 0.02 (0.10) |
| Alpha2 power | 0.04 (0.09) | 0.09 (0.10) | 0.10 (0.10) |
| Beta power | 0.04 (0.10) | -0.02 (0.10) | -0.05 (0.10) |
| Gamma power | -0.13 (0.10) | -0.07 (0.09) | -0.12 (0.09) |
|  |  |  |  |
| Functional connectivity |  |  |  |
| JPE_inv_ theta | -0.32 (0.09)*** | -0.17 (0.09)# | -0.21 (0.10)* |
| AEC-c alpha | -0.21 (0.09)* | -0.31 (0.09)*** | -0.25 (0.09)** |
| PLI theta | 0.03 (0.10) | 0.11 (0.09) | 0.20 (0.10)* |

All LMM models are corrected for age and sex. Values are standardized beta (SE). Δ Aβ-PET BP_ND_ was missing for *n* = 2. We scaled predictor and outcome variables within each LMM to enable comparison of effect sizes. *Δ* = annual change; **P* < 0.05; ***P* < 0.01; ****P* < 0.001; # *P* < 0.1

**
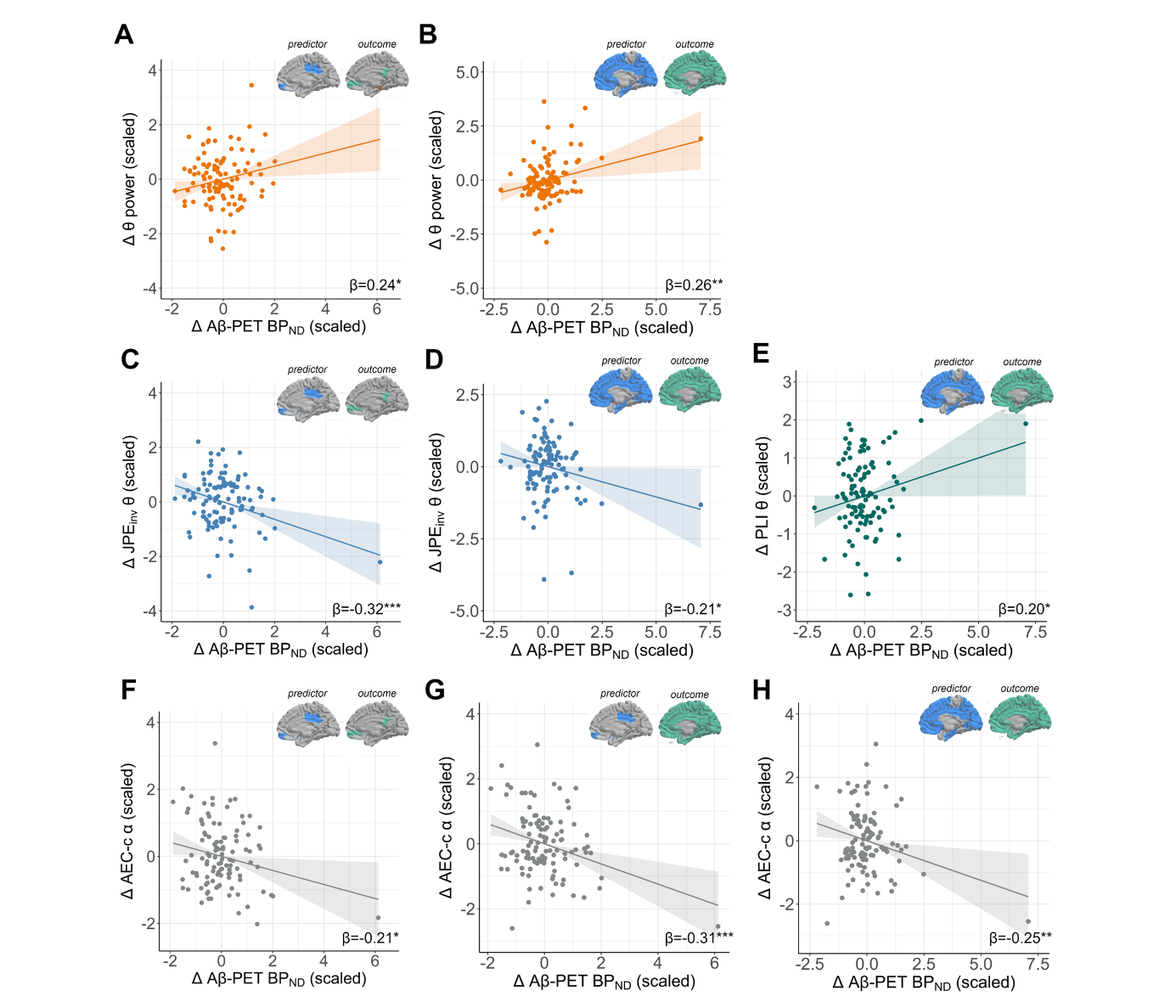
Supplementary Figure 1 Longitudinal associations between amyloid-β and MEG measures (including outlier)** Displayed are associations between z-transformed annual changes in Aβ BP_ND_ (x-axis) and z-transformed annual changes in MEG measures (y-axis). Changes in MEG measures were used as dependent variables in independent linear mixed models using change in Aβ BP_ND_ as independent variable and age and sex as covariates. Each data point represents an individual (*N* = 108). Annual change in Aβ BP_ND_ and theta power in the early AD ROI **(A)**. Annual change in whole-brain Aβ BP_ND_  and theta power **(B)**. Annual change in Aβ BP_ND_ in the early AD ROI and JPE_inv_ theta connectivity between the early AD ROI and the rest of the brain **(C)**. Annual change in whole-brain Aβ BP_ND_ and JPE_inv_ theta connectivity (**D**). Annual change in whole-brain Aβ BP_ND_ and PLI theta connectivity **(E)**. Annual change in Aβ BP_ND_ in the early AD ROI and AEC-c alpha connectivity between the early AD ROI and the rest of the brain **(F)**. Annual change in Aβ BP_ND_ in the early AD ROI and whole-brain AEC-c alpha connectivity **(G)**. Annual change in whole-brain Aβ BP_ND_ and AEC-c alpha connectivity **(H)**. Δ Aβ PET BP_ND_ was missing for *N* = 2. *Δ* = annual change; **P* < 0.05; ***P* < 0.01; ****P* < 0.001. Aβ, amyloid β; AEC-c, corrected amplitude envelope correlation; BP_ND_, non-displaceable binding potential; JPE_inv_, inverted joint permutation entropy; PET, positron emission tomography; PLI, phase lag index.

**
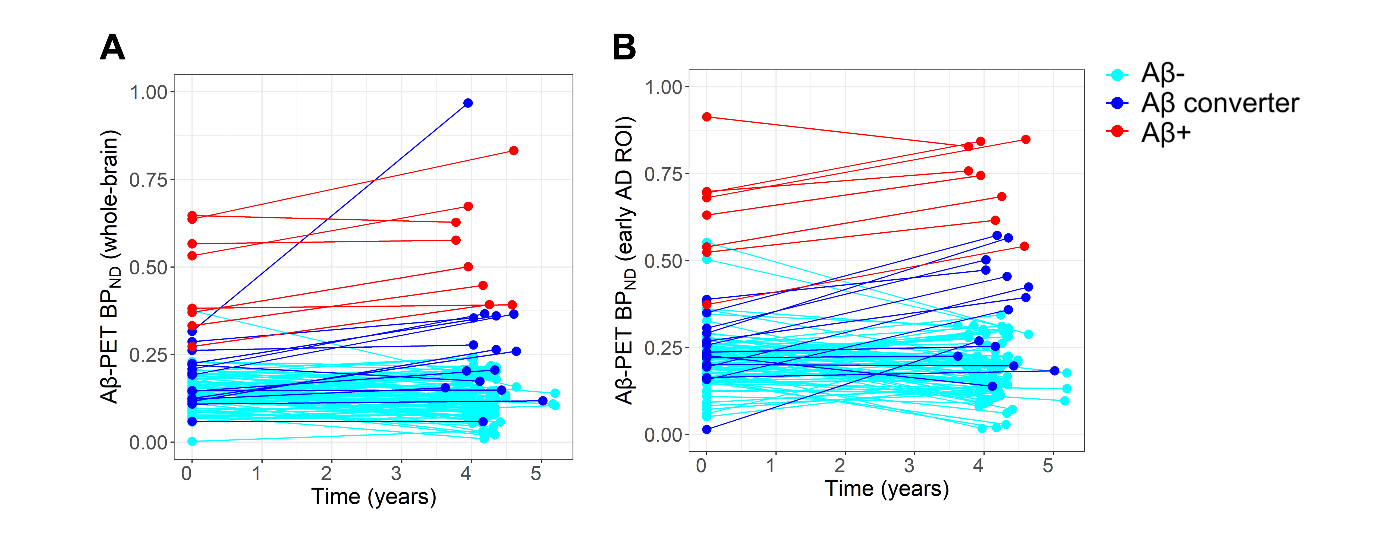
**

**Supplementary Figure 2 Longitudinal change in** **Aβ BP_ND_ for each participant between baseline and follow-up (unscaled).** The longitudinal change in Aβ BP_ND_ for each participant is depicted through two connected datapoints. The first datapoint, positioned at Time (years) = 0, corresponds to the participant’s Aβ BP_ND_ at the baseline visit, while the second datapoint represents the participant’s Aβ BP_ND_ at the follow-up visit. Longitudinal change on whole-brain level **(A)**. Longitudinal change within the early AD ROI **(B)**. Aβ-, amyloid-negative at both baseline and follow-up; Aβ converter, amyloid-negative at baseline and amyloid-positive at follow-up; Aβ+, amyloid-positive at both baseline and follow-up; AD, Alzheimer’s disease; BP_ND_, non-displaceable binding potential; PET, positron emission tomography; ROI, region of interest.

**
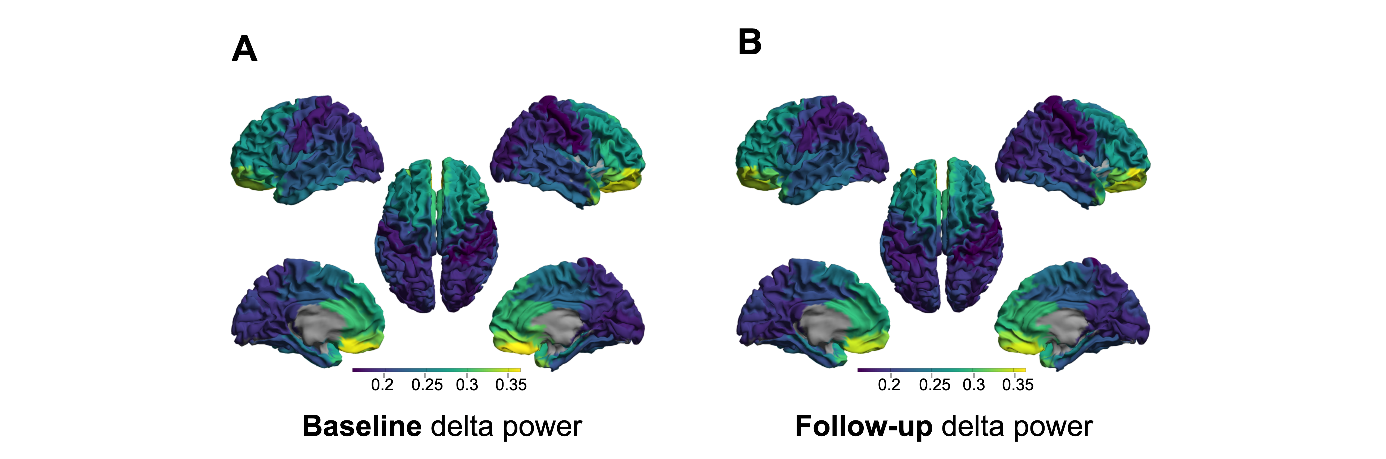
**

**Supplementary Figure 3 Cortical relative delta power distribution at baseline and follow-up, averaged across 110 subjects.** Relative delta power distribution at baseline **(A).** Relative delta power distribution at follow-up **(B)**. Visual inspection of the plots reveals highest relative delta power in the orbitofrontal gyri. This is likely driven by ocular artifacts.

**Supplementary Table 5** Associations between amyloid-β BP_ND_ in the early AD ROI and MEG measures in the posterior cingulate cortices

| Early AD ROI Aβ BP_ND_~ Posterior cingulate MEG | Outcome variable | Baseline and interaction effect with time | Standardized β (SE) | P-value |
| --- | --- | --- | --- | --- |
|  | Delta power | Aβ PET BP_ND_ | 0.03 | 0.65 |
|  |  | Aβ PET BP_ND_ x Time | -0.02 | 0.42 |

All LMM models are corrected for age and sex. Values are standardized beta (SE). Follow-up Aβ-PET BP_ND_ was missing for *n* = 2. We scaled predictor and outcome variables within each LMM to enable comparison of effect sizes.
